# COVID-19–Associated Hospitalizations Among U.S. Adults Aged ≥65 Years — COVID-NET, 13 States, January–August 2023

**DOI:** 10.15585/mmwr.mm7240a3

**Published:** 2023-10-06

**Authors:** Christopher A. Taylor, Kadam Patel, Monica E. Patton, Arthur Reingold, Breanna Kawasaki, James Meek, Kyle Openo, Patricia A. Ryan, Anna Falkowski, Erica Bye, Kelly Plymesser, Nancy Spina, Brenda L. Tesini, Nancy E. Moran, Melissa Sutton, H. Keipp Talbot, Andrea George, Fiona P. Havers, COVID-NET Surveillance Team, COVID-NET Surveillance Team, Pam Daily Kirley, Isaac Armistead, Kimberly Yousey-Hindes, Nadine Oosmanally, Maya L. Monroe, Justin Henderson, Paige D’Heilly, Emily B. Hancock, Grant Barney, Sophrena Bushey, Laurie M. Billing, Nasreen Abdullah, William Schaffner, Emma Mendez

**Affiliations:** ^1^Coronavirus and Other Respiratory Viruses Division, National Center for Immunization and Respiratory Diseases, CDC; ^2^General Dynamics Information Technology, Inc., Atlanta, Georgia; ^3^California Emerging Infections Program, Oakland, California; ^4^Colorado Department of Public Health & Environment; ^5^Connecticut Emerging Infections Program, Yale School of Public Health, New Haven, Connecticut; ^6^Emory University School of Medicine, Atlanta, Georgia; ^7^Georgia Emerging Infections Program, Georgia Department of Public Health; ^8^Atlanta Veterans Affairs Medical Center, Decatur, Georgia; ^9^Maryland Department of Health, Baltimore, Maryland; ^10^Michigan Department of Health & Human Services; ^11^Minnesota Department of Health; ^12^New Mexico Department of Health; ^13^New York State Department of Health; ^14^University of Rochester School of Medicine and Dentistry, Rochester, New York; ^15^Ohio Department of Health; ^16^Public Health Division, Oregon Health Authority, Portland, Oregon; ^17^Vanderbilt University Medical Center, Nashville, Tennessee; ^18^Salt Lake County Health Department, Salt Lake City, Utah.; California Emerging Infections Program; Colorado Department of Public Health & Environment; Connecticut Emerging Infections Program; Yale School of Public Health; Georgia Emerging Infections Program; Georgia Department of Public Health; Maryland Department of Health; Michigan Department of Health & Human Services; Minnesota Department of Health; University of New Mexico Emerging Infections Program; New York State Department of Health; University of Rochester School of Medicine and Dentistry; Ohio Department of Health; Public Health Division; Oregon Health Authority; Vanderbilt University Medical Center; Salt Lake County Health Department.

**Summary**

**What is already known about this topic?**

Adults aged ≥65 years have increased risk for COVID-19–associated hospitalization and other severe outcomes compared with younger age groups.

**What is added by this report?**

During January–August 2023, adults aged ≥65 years accounted for 62.9% of all COVID-19–associated hospitalizations. Most hospitalized adults aged ≥65 had multiple underlying conditions. Only 23.5% had received the recommended COVID-19 bivalent vaccine.

**What are the implications for public health practice?**

Adults with increased risk for COVID-19–associated hospitalization, including all adults aged ≥65 years, should reduce their risk for severe COVID-19 by receiving recommended COVID-19 vaccinations, adopting measures to reduce risk for contracting COVID-19, and seeking prompt outpatient antiviral treatment after a positive SARS-CoV-2 test result.

## Abstract

Adults aged ≥65 years remain at elevated risk for severe COVID-19 disease and have higher COVID-19–associated hospitalization rates compared with those in younger age groups. Data from the COVID-19–Associated Hospitalization Surveillance Network (COVID-NET) were analyzed to estimate COVID-19–associated hospitalization rates during January–August 2023 and identify demographic and clinical characteristics of hospitalized patients aged ≥65 years during January–June 2023. Among adults aged ≥65 years, hospitalization rates more than doubled, from 6.8 per 100,000 during the week ending July 15 to 16.4 per 100,000 during the week ending August 26, 2023. Across all age groups, adults aged ≥65 years accounted for 62.9% (95% CI = 60.1%–65.7%) of COVID-19–associated hospitalizations, 61.3% (95% CI = 54.7%–67.6%) of intensive care unit admissions, and 87.9% (95% CI = 80.5%–93.2%) of in-hospital deaths associated with COVID-19 hospitalizations. Most hospitalized adults aged ≥65 years (90.3%; 95% CI = 87.2%–92.8%) had multiple underlying conditions, and fewer than one quarter (23.5%; 95% CI = 19.5%–27.7%) had received the recommended COVID-19 bivalent vaccine. Because adults aged ≥65 years remain at increased risk for COVID-19–associated hospitalization and severe outcomes, guidance for this age group should continue to focus on measures to prevent SARS-CoV-2 infection, encourage vaccination, and promote early treatment for persons who receive a positive SARS-CoV-2 test result to reduce their risk for severe COVID-19–associated outcomes.

## Introduction

Since March 2020, population-based rates of COVID-19–associated hospitalization among all age groups have been highest among adults aged ≥65 years, with increasing age associated with higher hospitalization rates (*1*). During January–June 2023, rates of COVID-19–associated hospitalizations among all adults aged ≥18 years declined, including among adults aged ≥65 years. However, rates remained elevated among adults aged ≥65 years relative to younger age groups and increased beginning the week ending July 15, 2023.* Understanding the characteristics of this population who remain at increased risk for COVID-19–associated hospitalization can help guide appropriate prevention recommendations.

## Methods

COVID-NET^†^ conducts population-based surveillance for laboratory-confirmed COVID-19–associated hospitalizations among catchment area residents in 98 counties and across 13 U.S. states.^§^ COVID-19–associated hospitalizations are defined as those among persons who have received a positive SARS-CoV-2 reverse transcription–polymerase chain reaction (RT-PCR) or rapid antigen detection test result during or within the 14 days preceding hospitalization.

This analysis describes overall and age-stratified weekly COVID-19–associated hospitalization rates during January–August 2023, focusing on adults aged ≥65 years; rates (hospitalizations per 100,000 population) include all COVID-19–associated hospitalizations. Using previously described methods (*2*), trained surveillance officers abstracted demographic and clinical data from the medical charts of an age- and site-stratified random sample of hospitalized adults; data on sampled cases were available for January 1–June 30, 2023. Analyses of sampled cases were limited to hospitalizations for which COVID-19–related illness was the likely presenting complaint, based on information in the admission history and physical examination or face sheet^¶^ of the medical record at the time of admission.** Patient vaccination status (unvaccinated [received no COVID-19 vaccine], partially vaccinated, received monovalent vaccine only, or received ≥1 bivalent dose since September 2022),^††^ was obtained from state immunization information systems. Underlying conditions were chronic or preexisting medical conditions present at or before hospital admission.

Unweighted case counts and weighted percentages that better represent the hospitalized population of the catchment area (*2*) are presented for sampled data. Data were analyzed using SAS (version 9.4; SAS Institute); variances were estimated using Taylor series linearization method. Statistical differences between groups were assessed using chi-square tests; p<0.05 were considered statistically significant. This activity was reviewed by CDC, deemed not research, and was conducted in accordance with applicable federal law and CDC policy.^§§^

## Results

**FIGURE 1:**
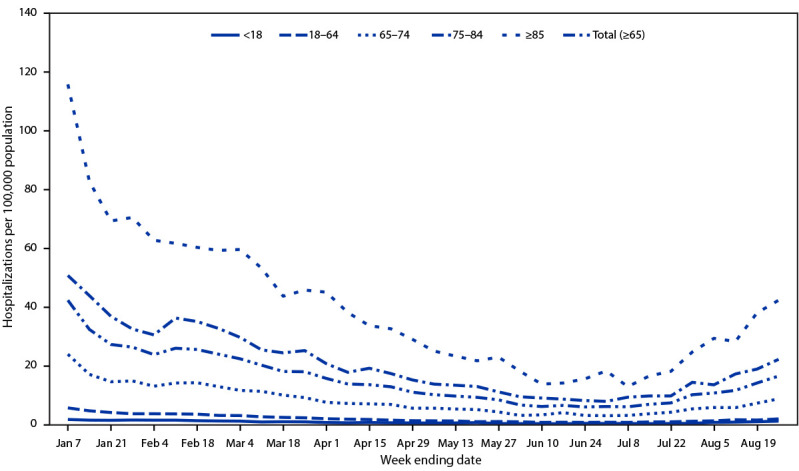
Weekly COVID-19–associated hospitalization* rates, by age group — COVID-NET, 13 states, January 1–August 26, 2023 **Abbreviation**: COVID-NET = COVID-19–Associated Hospitalization Surveillance Network. * COVID-19–associated hospitalizations among patients who received a positive SARS-CoV-2 test result during hospitalization or ≤14 days before admission.

During January 1–July 8, 2023, weekly rates of COVID-19–associated hospitalization among adults aged ≥65 years decreased 86%, from a high of 42.2 to 5.9 per 100,000, the lowest level since July 2021. Rates then increased to 6.8 for the week ending July 15 and continued to increase during subsequent weeks, to 16.4 for the week ending August 26, 2023 (Figure 1); during that week, the rate among adults aged ≥65 years was nine times as high as that among adults aged 18–64 years (1.8) and 16 times as high as that among persons aged <18 years (1.0). Among adults aged ≥65 years, COVID-19–associated hospitalization rates were highest among those aged ≥85 years (42.2) and lowest among adults aged 65–74 years (8.6).

Adults aged ≥65 years accounted for 62.9% of all COVID-19–associated hospitalizations during January–August 2023, a one third increase from 45.9% during March 2020–December 2022 (p<0.01) (Supplementary Figure 1; https://stacks.cdc.gov/view/cdc/133298). Persons aged ≥65 years accounted for 62.8% (95% CI = 60.1%–65.7%) of the 4,232 COVID-19–associated hospitalizations sampled during January–June 2023, for 61.3% (95% CI = 54.7%–67.6%) of all intensive care unit (ICU) admissions, and for 87.9% (95% CI = 80.5%–93.2%) of in-hospital deaths occurring during COVID-19–associated hospitalizations.

**FIGURE 2:**
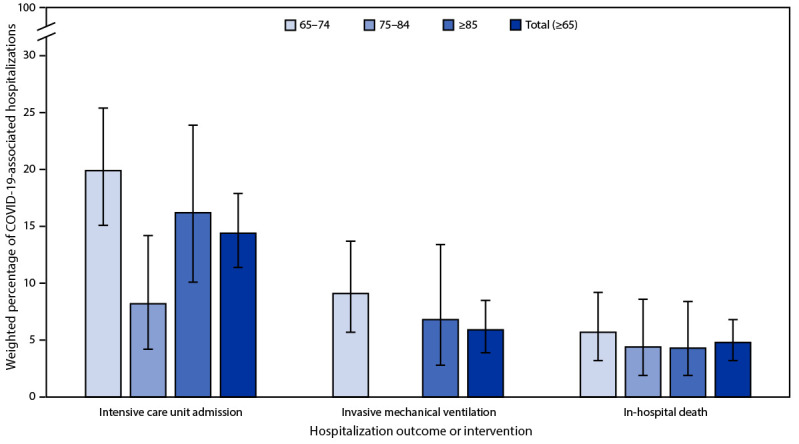
Percentage*^,^^†^^,^^§^^,^^¶^ of adults aged ≥65 years with laboratory-confirmed SARS-CoV-2 infection for which COVID-19–related illness was the likely presenting complaint with severe hospitalization interventions and outcomes, by age group — COVID-NET, 13 states, January–June 2023 **Abbreviation**: COVID-NET = COVID-19–Associated Hospitalization Surveillance Network. * With 95% CIs indicated by error bars. ^†^ Proportions are from a weighted sample of hospitalized adults with completed medical chart abstraction and a discharge disposition. ^§^ Data are not presented when sample size <10 (invasive mechanical ventilation for persons aged 75–84 years). ^¶^ Relative SEs for estimated percentages of in-hospital deaths among patients aged 75–84 and ≥85 years and for invasive mechanical ventilation among persons aged ≥85 years are >30, and therefore, estimates might be unstable because of small sample sizes.

Of the 1,465 adults sampled from among the 21,445 COVID-19–associated hospitalizations in persons aged ≥65 years, COVID-19 was the likely presenting complaint upon admission for 1,133 (79.5%; 95% CI = 75.9%–82.9%) patients who were included in analyses of clinical data (Table).^¶¶^ Among these patients, 31.0%, were aged 65–74 years, 36.5% were aged 75–84 years, and 32.5% were aged ≥85 years; overall, 202 (17.7%) patients were residents of a long-term care facility (LTCF). Among the sampled patients, 176 (14.4%) were admitted to an ICU, 68 (5.9%) received invasive mechanical ventilation, and 63 (4.8%) patients died during the hospitalization (Figure 2); these proportions did not differ substantially among subgroups of hospitalized patients aged ≥65 years (p>0.05 for all).

**TABLE:** Demographic characteristics and clinical outcomes of hospitalized adults aged ≥65 years with laboratory-confirmed SARS-CoV-2 infection with COVID-19–related illness as the likely presenting complaint,* by age group — COVID-NET, 13 states, January–June 2023

Characteristic	Age group, yrs, no. (%) [95% CI]			
	Total (≥65)	65–74	75–84	≥85
**Total**	**1,133 (100)**	**544 (31.0) [27.1–35.0]**	**289 (36.5) [31.6–41.6]**	**300 (32.5) [28.0–37.4]**
**Sex**				
Female	**544 (48.8) [44.0–53.7]**	277 (49.8) [43.5–56.0]	121 (42.1) [33.3–51.3]	146 (55.4) [46.3–64.2]
Male	**(79.1) [72.2–86.0]**	(68.9) [66.4–71.6]	(87.9) [85.0–90.8]	(78.4) [76.4–80.8]
**Race and ethnicity^†^**				
A/PI	**43 (4.7) [2.6–7.9]**	25 (6.0) [2.6–11.4]^§^	11 (5.9) [1.7–14.1]^§^	— ^¶^
Black or African American	**120 (14.4) [11.3–17.9]**	69 (18.1) [13.5–23.3]	22 (12.1) [6.7–19.5]	29 (13.6) [7.8–21.4]
White	**851 (68.6) [63.9–73.1]**	390 (64.7) [58.3–70.7]	230 (68.9) [58.8–77.8]	231 (72.1) [63.1–80.0]
Hispanic or Latino	**85 (7.8) [5.1–11.3]**	47 (8.0) [5.2–11.6]	17 (8.7) [3.2–18.3]^§^	21 (6.5) [3.1–11.8]^§^
All other races**	**21 (2.3) [1.0–4.8]**	— ^¶^	— ^¶^	— ^¶^
Unknown race	**13 (2.1) [0.9–4.1]**	— ^¶^	— ^¶^	— ^¶^
**Resident of long-term care facility**				
Yes	**202 (17.7) [14.3–21.5]**	66 (11.6) [8.0–16.2]	84 (25.5) [18.1–34.2]	52 (14.7) [10.0–20.6]
No	**931 (82.3) [78.5–85.7]**	478 (88.4) [83.8–92.0]	205 (74.5) [65.8–81.9]	248 (85.3) [79.4–90.0]
**Hospitalization intervention or outcome**				
Length of stay, days, median (IQR)	**3.9 (2.2–7.7)**	4.3 (2.1–9.4)	3.8 (2.5–6.9)	3.7 (2.1–7.1)
ICU admission	**176 (14.4) [11.4–17.9]**	105 (19.9) [15.1–25.4]	26 (8.2) [4.2–14.2]	45 (16.2) [10.1–23.9]
IMV	**68 (5.9) [3.9–8.5]**	48 (9.1) [5.7–13.7]	— ^¶^	16 (6.8) [2.8–13.4]^§^
In-hospital death	**63 (4.8) [3.2–6.8]**	31 (5.7) [3.2–9.2]	17 (4.4) [1.9–8.6]^§^	15 (4.3) [1.9–8.4]^§^

**Abbreviations:** A/PI = Asian or Pacific Islander; COVID-NET = COVID-19–Associated Hospitalization Surveillance Network; NH = non-Hispanic; ICU = intensive care unit; IMV = invasive mechanical ventilation.

* The likely presenting complaint upon admission is identified by trained COVID-NET surveillance officers using information in the admission history and physical or face sheet (one-page summary of the patient’s important information) of the medical record. The likely presenting complaint is identified and categorized as COVID-19–related illness; inpatient surgery or procedures; psychiatric admission requiring acute medical care; trauma; other (with an accompanying free-text field); or unknown. CDC clinicians independently review the free-text field of complaints classified as other to determine if the complaint might be recategorized or remain in the other category (e.g., skin and soft tissue infections). Hospitalizations for which the likely primary complaint was not COVID-19–related illness, including those for which the likely presenting complaint was unknown or remained classified as other, were categorized as having a presenting complaint not likely related to COVID-19.

^†^ If ethnicity was unknown, NH ethnicity was assumed. Persons of Hispanic or Latino (Hispanic) origin might be of any race but are categorized as Hispanic; all racial groups are NH.

^§^ Relative SE >30; estimates might be unstable because of small sample size.

^¶^ Data are not presented for cells with sample size <10.

** Includes NH American Indian or Alaska Native and multiracial persons.

Nearly all sampled patients (1,112 [98.5%; 95% CI = 97.4%–99.2%]) had at least one underlying condition and most (1,015 [90.3%; 95% CI = 87.2%–92.8%]) had two or more. The most common specific conditions reported included diabetes (39.0%; 95% CI = 34.2%–43.9%), kidney disorders (28.0%; 95% CI = 23.8%–32.5), coronary artery disease*** (27.4%; 95% CI = 23.3%–31.8%), chronic heart failure or cardiomyopathy (26.1%; 95% CI = 22.1%–30.4%), and obesity (25.6%; 95% CI = 21.5%–30.1%) (Supplementary Figure 2; https://stacks.cdc.gov/view/cdc/133298).

Overall, 159 (15.9%; 95% CI = 12.4%–19.9%) of sampled patients aged ≥65 years had not been vaccinated against COVID-19 at the time of hospital admission, 703 (58.6%; 95% CI = 53.7%–63.4%) had received only a monovalent vaccine, and 240 (23.5%; 95% CI = 19.5%–27.7%) had received ≥1 bivalent COVID-19 dose (Supplementary Figure 3, https://stacks.cdc.gov/view/cdc/133298).

## Discussion

During January–mid-July 2023, COVID-19–associated hospitalization rates among persons aged ≥65 years declined but then increased through the week ending August 26, 2023. Throughout the same period, adults aged ≥65 years continued to have the highest hospitalization rates of any age group, accounting for approximately one half of all COVID-19–associated hospitalizations and ICU admissions as well as nearly 90% of in-hospital deaths. Most adults aged ≥65 years who were hospitalized with a positive SARS-CoV-2 test result were likely admitted because of COVID-19 illness and, among these, a substantial proportion had severe outcomes, including ICU admission, receipt of invasive mechanical ventilation, and in-hospital death. Approximately one in six adults aged ≥65 years hospitalized for COVID-19 were LTCF residents. These findings suggest that COVID-19–associated hospitalization continues to predominantly affect adults aged ≥65 years and represent a continued public health threat.

Nearly all hospitalized adults aged ≥65 years had two or more underlying medical conditions. A previous COVID-NET analysis found that adults with two or more underlying medical conditions had a greater than fourfold increased risk for hospitalization after adjusting for age, sex, and race and ethnicity (*3*). Although asymptomatic or mildly ill patients with positive SARS-CoV-2 test results might be hospitalized for non-COVID reasons, based on information in the admission history and physical examination or face sheet of the medical record, approximately three quarters of hospitalized adults aged ≥65 years in this analysis were likely admitted primarily for COVID-19–related illness, which caused substantial morbidity and mortality in this age group.

In September 2022, the Advisory Committee on Immunization Practices (ACIP) recommended a bivalent COVID-19 vaccine dose (*4*) and in April 2023, recommended ≥1 additional bivalent dose for adults aged ≥65 years (*5*). However, this analysis found that approximately three quarters (76.5%) of adults aged ≥65 years hospitalized for COVID-19 during January–June 2023 had not received a bivalent dose, and 16% had not received any COVID-19 vaccine. Although bivalent vaccine effectiveness against COVID-19–associated hospitalization has been shown to decline over time, effectiveness in preventing hospitalization and severe outcomes, such as ICU admission, has been documented (*6*). ACIP recently recommended that all persons aged ≥6 months, including those aged ≥65 years, receive an updated (2023–2024 Formula) COVID-19 vaccine for the 2023–2024 respiratory season (*7*). In addition to vaccination and adoption of measures to reduce risk for contracting SARS-CoV-2, other strategies shown to reduce COVID-19–associated hospitalization risk include early outpatient treatment with ritonavir-boosted nirmatrelvir (Paxlovid), remdesivir (Veklury), or molnupiravir (Lagevrio) for persons with SARS-CoV-2 infection who are at high risk for progression to severe disease, including all adults aged ≥65 years (*8*,*9*). Prevention, vaccination, and early antiviral treatment are important tools in preventing hospitalization and severe associated outcomes in this high-risk age group.

### Limitations

The findings in this report are subject to at least three limitations. First, COVID-19–associated hospitalizations might have been missed because of hospital testing practices or test availability, and therefore, hospitalization rates might be underestimated. Second, a patient’s likely presenting complaint at the time of admission is subject to misclassification and might have resulted in cases being unintentionally included or excluded from this analysis. Hospitalization records that do not specify COVID-19 or respiratory illness as a likely presenting complaint can still result in COVID-19–related illness and might affect clinical decision-making and the course of hospitalization. Finally, the COVID-NET catchment areas include approximately 10% of the U.S. population; thus, these findings might not be nationally generalizable.

### Implications for Public Health Practice

COVID-19–associated hospitalization rates declined among persons of all ages during January–July 2023 but increased starting in mid-July 2023. Rates among adults aged ≥65 years remained higher than those among younger age groups, and this older age group accounted for approximately 60% of all COVID-19–associated hospitalizations and nearly 90% of deaths during hospitalization. Many hospitalized adults aged ≥65 years had multiple underlying medical conditions, and most had not received the COVID-19 bivalent vaccine, which had been recommended before the period of this analysis.

COVID-19–associated hospitalizations continue to predominantly affect adults aged ≥65 years and represent a continued public health threat. All adults, especially those aged ≥65 years and others at high risk for progression to severe COVID-19 illness,^†††^ should reduce their risk for COVID-19–related hospitalizations and severe outcomes by receiving recommended COVID-19 vaccines, adopting measures to reduce risk for contracting SARS-CoV-2,^§§§^ and seeking early outpatient antiviral treatment after receipt of a positive SARS-CoV-2 test result.

## Footnotes

*
https://www.cdc.gov/coronavirus/2019-ncov/covidnetdashboard/de/powerbi/dashboard.html

† COVID-NET is one of three platforms comprising the Respiratory Virus Hospitalization Surveillance Network (RESP-NET). These platforms conduct population-based surveillance for hospitalization associated with respiratory syncytial virus (RSV) (RSV-NET), COVID-19 (COVID-NET) and influenza (FluSurv-NET). https://www.cdc.gov/surveillance/resp-net/dashboard.html

§ Selected counties in California, Colorado, Connecticut, Georgia, Maryland, Michigan, Minnesota, New Mexico, New York, Ohio, Oregon, Tennessee, and Utah. https://www.cdc.gov/mmwr/volumes/69/wr/mm6915e3.htm

¶ A one-page summary of important information about a patient.

** The likely presenting complaint upon admission is identified by trained COVID-NET surveillance officers using information in the admission history and physical examination or face sheet of the medical record. The likely presenting complaint is identified and categorized as 1) COVID-19–related illness, 2) inpatient surgery or procedures, 3) psychiatric admission requiring acute medical care, 4) trauma, 5) other (with an accompanying free-text field), or 6) unknown. CDC clinicians independently reviewed the free-text field of complaints classified as other to ascertain whether the complaint might be recategorized or remain in the other category (e.g., skin and soft tissue infections). Hospitalizations for which the likely primary presenting complaint was not COVID-19–related illness, including those for which the likely presenting complaint was unknown or remained classified as other, were categorized as having a presenting complaint not likely related to COVID-19.

†† Adults who had no record of receiving any COVID-19 vaccination were considered unvaccinated. Adults who started but did not complete a primary series ≤14 days before a positive test result for SARS-CoV-2 associated with their hospitalization were considered partially vaccinated. Adults who completed the primary COVID-19 vaccination series, including receipt of the second dose of a 2-dose primary vaccination series or a single dose of a 1-dose primary vaccine product ≥14 days before receipt of a positive SARS-CoV-2 test result associated with their hospitalization, regardless of any additional monovalent doses, but who received no bivalent doses, were considered to have received monovalent vaccine only. Adults vaccinated with ≥1 bivalent dose, regardless of primary vaccination series status, received ≥14 days before a positive test result for SARS-CoV-2 associated with their hospitalization, were considered to have received ≥1 bivalent dose. CDC first recommended vaccination of adults aged ≥65 years in September 2022, followed by a subsequent recommendation in April 2023 for adults aged ≥65 years to receive ≥1 additional bivalent dose.

§§ 45 C.F.R. part 46, 21 C.F.R. part 56; 42 U.S.C. Sect. 241(d); 5 U.S.C. Sect. 552a; 44 U.S.C. Sect. 3501 et seq.

¶¶ Among the 321 (20.0%) patients with other likely primary reasons for admission, 31 (2.5%) were admitted for trauma, 25 (2.0%) for planned surgery or procedure, 16 (<1%) for psychiatric admissions requiring acute medical care, and 243 (14.2%) with other reasons for admission, which included acute infections (e.g., abscess) likely not COVID-19–related. Admissions categorized as other were reviewed independently by at least two physicians using a standardized algorithm. Fewer than 10 hospitalized patients (<1%) had an unknown reason for admission.

*** Includes history of coronary artery bypass graft or myocardial infarction.

†††
https://www.cdc.gov/coronavirus/2019-ncov/hcp/clinical-care/underlyingconditions.html

§§§
https://www.cdc.gov/coronavirus/2019-ncov/prevent-getting-sick/prevention.html
